# How Do We Connect Brain Areas with Cognitive Functions? The Past, the Present and the Future

**DOI:** 10.3390/neurosci3030037

**Published:** 2022-09-14

**Authors:** Khushboo Verma, Satwant Kumar

**Affiliations:** 1 Department of Neurology, Dell Medical School, The University of Texas, Austin, TX 78712, USA; 2 Center for Perceptual Systems, University of Texas, Austin, TX 78712, USA

**Keywords:** cognition, cognitive functions, localization, lesion studies, body perception, functional magnetic resonance imaging (fMRI), electrical microsimulation, transcranial magnetic stimulation, extrastriate body area, fusiform body area

## Abstract

One of the central goals of cognitive neuroscience is to understand how structure relates to function. Over the past century, clinical studies on patients with lesions have provided key insights into the relationship between brain areas and behavior. Since the early efforts for characterization of cognitive functions focused on localization, we provide an account of cognitive function in terms of localization. Next, using body perception as an example, we summarize the contemporary techniques. Finally, we outline the trajectory of current progress into the future and discuss the implications for clinical and basic neuroscience.

## 1. Introduction

### 1.1. Characterizing Cognition

Characterization of cognitive functions is the ultimate challenge for cognitive neuroscientists and neurologists. Characterization begins with the definition of the function, and its precise localization in the brain. Cognitive or executive control refers to the ability to accomplish a goal-directed task, that is, to direct attention to the task, to inhibit conflicting distractions, to self-monitor, and to use working memory efficiently to complete the task [1]. This definition of executive control encompasses multiple cognitive domains such as attention, inhibition, and working memory. It is imperative to define and localize the individual domains to completely characterize cognition. However, it is unclear if these individual cognitive domains are diffusely located over the cortex or if they are discreetly localized. The difficulties with localization exist because testing an individual domain with a task does not guarantee the isolation of that particular cognitive domain. This referred to as the ‘task impurity issue’ [1]. Although the individual cognitive functions and domains can be differentiated from one another, they share a commonality that prevents them from being discretely characterized. This concept of the coexistence of unity and diversity was first proposed by Teuber [2] and was empirically studied by Miyake et al. [3]. Considering these challenges, we propose utilizing a theoretical framework to conceptualize the comprehensive characterization of cognitive functions.

### 1.2. Theoretical Model

**Figure 1:**
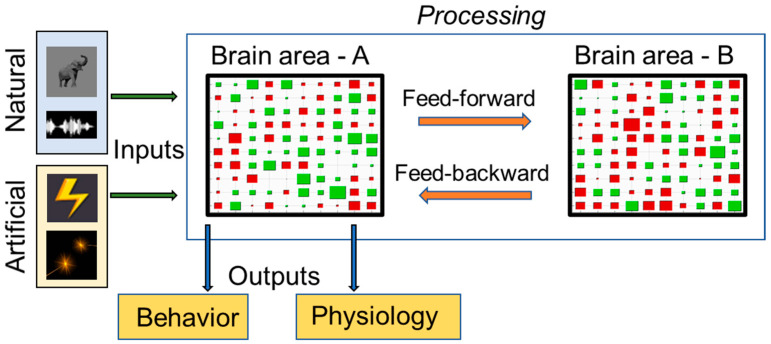
Envisioning Marr’s theoretical framework for characterization of a given brain area (Brain area—A) as a sensory information processing system. At the computational level, the relevance of the processing can be visualized as the system taking in inputs and generating outputs relevant to the organism. The inputs are shown on the extreme left as natural (visual and auditory) and artificial (electrical microsimulation and optogenetic stimulation) stimuli. Outputs are shown as behavioral and physiological manifestations. This framework also considers the relative compositions of areas A and B with 2 subtypes of neurons, that is excitatory (indicated as green rectangles), and inhibitory (indicated as red rectangles). Additionally, at an algorithmic level, processing as shown in the light blue box considers both feed-forward and feed-backward communications and relative firing rates of neurons (indicated with sizes of red and green rectangles).

Marr provided a 3-level description to understand an information processing system that can be used to characterize cognitive functions [4]. The initial level is computational, which entails what is being computed and the relevance of the computational process. Therefore, this level describes why a process occurs. Second, there is the algorithmic level, which describes how a process occurs. This level includes information about the input and output and the algorithmic transformation from input to output. The third level deals with the implementation of the process, that is how a process is being conducted [5]. Figure 1 illustrates how these theoretical levels can be applied to a cognitive function.

**Figure 2:**
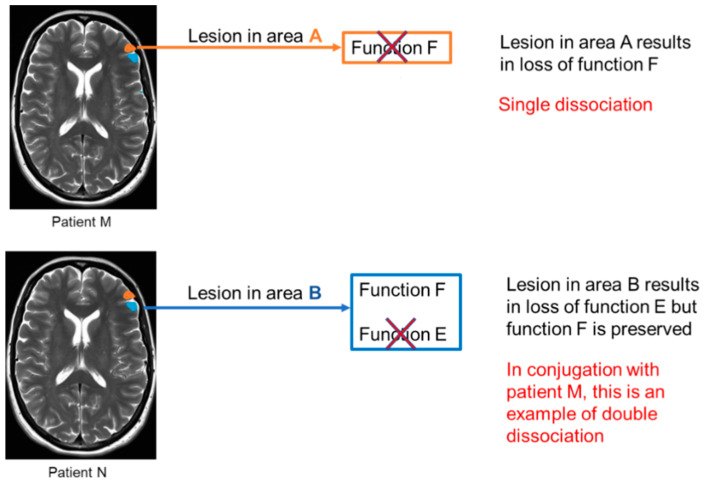
Schematic representation of single and double dissociation. In this example a lesion in area A (marked with orange in the MR image) leads to a loss of function F; however, function E is preserved. This represents single dissociation. In patient N, there is a lesion in area B (marked with blue in MR image), which leads to loss of function E however function F is preserved. This in conjugation with patient M who has a loss of function F but preserved function E, represents an example of double dissociation.

In the question pertaining to this review, “How are brain areas connected to cognitive functions?”, connection refers to the essential evidence required to establish a link between a brain area and a particular cognitive function. This essential evidence entails the necessity and functional specificity of a brain area. Necessity implies the causal role of the brain area in the cognitive function that is, if there is a lesion in an area it will lead to the loss of that cognitive function. This is an example of a single dissociation study. When this concept is applied to two separate subjects with different lesion locations and loss of separate functions, it is termed as double dissociation. When these two cases are considered in conjugation there is a higher degree of confidence that these two functions are modular and are non-overlapping in location [6]. Figure 2 demonstrates this concept.

In this review, we discuss how characterization of cognitive functions has been approached in the past. We also explore the ongoing efforts, and their future implications. Since the early efforts for characterization focused on localization, we give an account of cognitive function in terms of localization. To describe the present efforts, we have chosen a perceptual-cognitive function body perception [7]. Taking body perception as an example, we summarize contemporary techniques. We ask questions throughout the narrative and seek answers together with the readers to evoke curiosity.

## 2. The Past

The journey begins with Franz Gall in the late eighteenth century. He encountered patients with certain neurological deficits; however, neuroimaging was not available to visualize the brain in vivo. He postulated that personality traits and aspects of cognition such as language are localized in different regions of the cerebrum. Moreover, he hypothesized that this information can be inferred from the bumps on the skull. Although there is a lack of scientific rigor in this approach, this was one of the first attempts at proposing the concept of localization of cognitive functions. The theory also implies that distinct subparts of the brain perform specific functions independently [8]. 

Were there any techniques available to test Gall’s hypothesis experimentally? One possibility was to create artificial lesions in animals and study the effects. This technique was utilized by Pierre Flourens, a French physician and anatomist. Since the neurosurgical instruments at this time were not sophisticated to create precise lesions, he did not find specific cognitive deficits with the discrete lesions. Consequently, he removed different parts of the brain and examined the results. When the cerebellum was removed there was a loss of coordination and balance. Similarly, when the cerebrum was removed there was a loss of motor function and judgment. Based on these experiments he concluded that cognitive functions are diffusely located all over the cerebrum [9].

**Figure 3:**
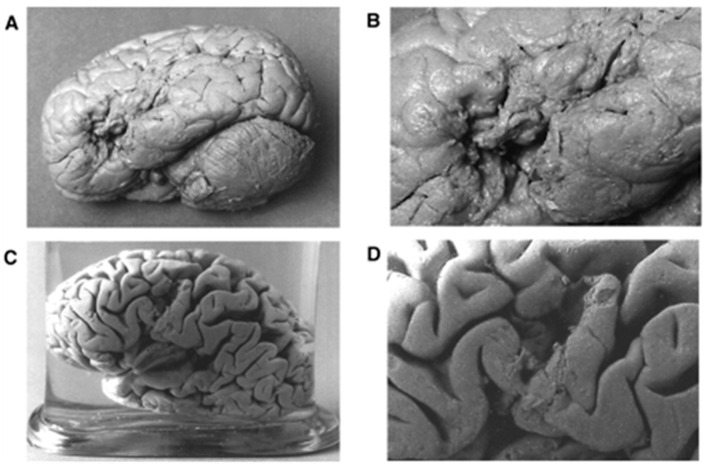
Gross anatomical features of brains of Broca’s patients, L1 (nicknamed Tan) and L2 with aphasia. Panels (**A**,**C**) are the lateral views of patients L1′s and L2′s brain. Panels (**B**,**D**) are the close-up of views of the brain lesions for the patients L1 and L2, respectively. Panels (**A**) through (**D**) are adapted with permission from Dronkers et al. (2007).

Since this was conducted in animals, one gets curious and asks: *Was there a way to look at the brains of patients with specific cognitive deficits?* Paul Broca wondered the same when he met a patient famously nicknamed Tan. This patient could understand what was spoken to him, was able to gesture to communicate, and had intact emotional responses; however, he had no spontaneous speech except the word tan. After the patient’s death, Broca performed an autopsy and discovered a lesion in the left frontal lobe. He concluded that the patient’s speech deficit was due to this lesion [10]. Figure 3 shows the gross anatomical findings of Broca’s two patients with expressive aphasia and lesions in similar locations. 

By concluding that these lesions were responsible for the language deficits, Broca concurred with Gall’s hypothesis of localization. He additionally proposed that the major anatomical sulci and gyri are not arbitrary: rather, they divide the brain into lobes that subserve specific roles [10]. A major drawback of his method was that an autopsy was required to study the brain. Consequently, the sample size was inadvertently constrained by the inability to follow patients for life [11].

Given the limitations with human lesions studies, one asks: *Could smaller lesions be created in animals to test the hypothesis of localization?* Two German scientists, Gustav Fritsch and Eduard Hitzig, performed electrical stimulation of the awake dogs and noticed that the stimulation of specific cortical sites led to extension or flexion movements of the front or rear paws. This was considered potential evidence for localization and sparked a debate in the scientific community, which subsequently led to further experiments to confirm the findings [12].

**Figure 4:**
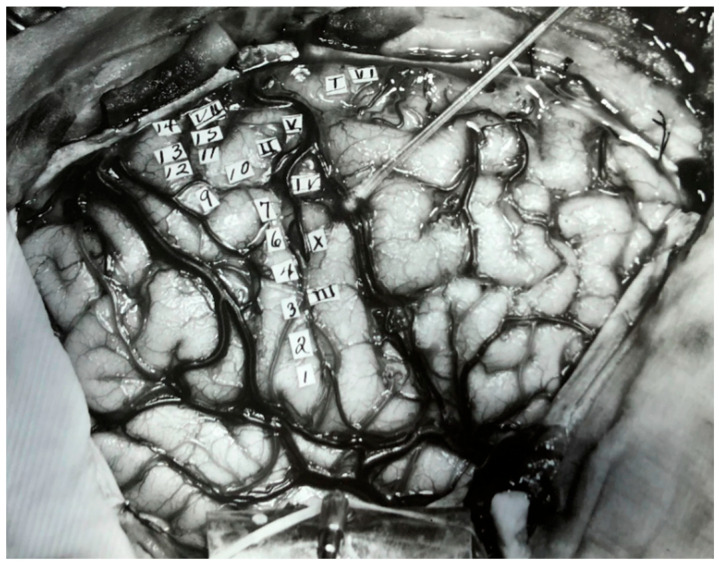
Regions in the right hemisphere indicated with Roman and Arabic numeral labels. The Roman numerals represent sites where electrical stimulation led to sensory perception, and the Arabic numerals indicate sites where stimulation lead to motor responses. Figure adapted with permission from Leblanc (2021).

This leads to the questions: *Was electrical stimulation of human brains possible and would similar effects be observed?* Wilder Penfield, a neurosurgeon who operated on patients with intractable epilepsy and tumors, considered this question. He and Edwin Boldery meticulously studied the motor and sensory cortex by electrically stimulating specific areas of the cortex [13]. Figure 4 is an operative photograph of one such patient. 

**Figure 5:**
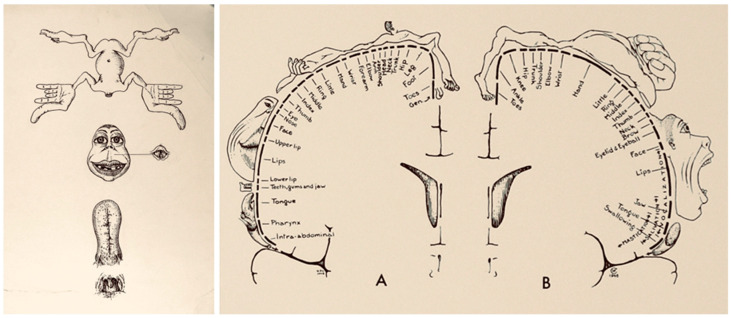
Evolution of homunculus. The illustration on the left is the 1937 homunculus, and the illustration on the right is the 1950 homunculus. The 1937 homunculus had a single figurine accounting for sensorimotor function. Illustrations adapted with permission from Leblanc (2021). The figurine is divided into four parts: body, face, tongue, and nasopharynx. The 1950 homunculus has distinct figurines showing the sensory and motor homunculus labelled as (**A**,**B**), respectively.

Their work led to the creation of motor and sensory homunculi, which are still widely used by neurologists to ascertain localization of motor and sensory deficits [13]. Figure 5 illustrates the evolution of homunculi from 1937 to 1950.

Homunculi are widely used as they enable a simple representation of somatotropic organization; however, they have certain limitations. First, the regions do not represent precise organization between the cortical regions and the individual muscles or muscle groups. This is illustrated with the concept of convergence and divergence. Convergence stands for specific motor activation which can be achieved by more than one cortical site activation. Divergence on the other hand stands for the activation of multiple muscle groups by stimulation at a single site [14]. Furthermore, they do not account for the concept of negative representation, that is inhibition of muscle movement due to stimulation at a site [15]. 

## 3. The Present

Now, we will embark on an exciting journey into contemporary neuroscience. We will study the advancement of our understanding of a specialized cognitive function: body perception. This is a unique cognitive function that encompasses a wealth of social information. In addition to age, sex, and identity of an individual, it includes abstract elements such as intention and emotion [7].

**Figure 6:**
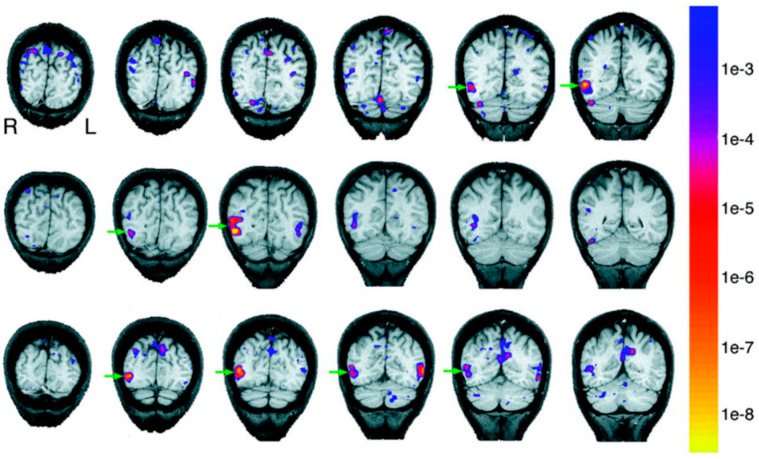
Coronal sections of 3 subjects arranged in separate rows. The anatomical slices are overlaid with statistical maps of voxels with selective response for human bodies and body parts. Adapted with permission from Downing et al. (2001). The green arrow indicates the region with maximal response. The color scale on the right stands for the *p* values of the activations.

The first question we ask is: *Do we have discrete areas for body perception?* In 2001, Downing and Kanwisher explored this using functional magnetic resonance imaging (fMRI). In this study, subjects viewed images of human bodies while undergoing fMRI. A specific area in the right lateral occipitotemporal cortex was noted to be selectively responsive to bodies and body parts excluding the face [16]. This area is shown in coronal slices with green arrow in Figure 6. 

**Figure 7:**
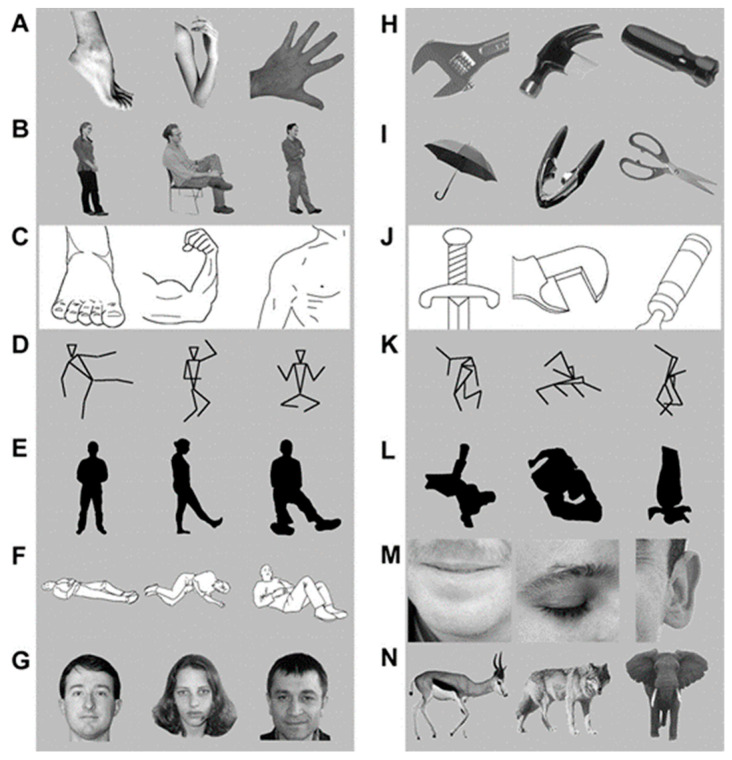
The range of stimuli included in the study, with various depictions of bodies such as body parts (**A**), complete human bodies (**B**), line drawings of body parts (**C**), stick figure (**D**), silhouettes (**E**), and images with inferred motion (**F**). The response was high in all these stimuli as compared to the low response for stimuli H through N. These stimuli are object parts (**H**), complete objects (**I**), line drawings of objects (**J**), scrambled stick figures (**K**), and scrambled silhouettes (**L**). The responses were intermediate for human faces (**G**), face parts (**M**), and mammals (**N**). Panels (**A**–**N**) adapted with permission from Downing et al. (2001).

However, if we consider all possibilities, multiple alternative explanations exist. A few examples being: What if this area is responsive to low level features of visual stimuli, such as shades and textures? What if this area responds to any objects or bodies of any animal? What if this area responds to anything human, such as human faces? All these questions refer to the selectivity of response to the stimulus of interest (in this case bodies), and a single question that encompasses all the above questions is, *does this region selectively respond to bodies?* To answer this, Downing and Kanwisher included multiple stimuli belonging to diverse categories, which is depicted in Figure 7. 

In this experiment, the response to these control stimuli was studied solely in the region that had initially responded to the bodies. The authors found that this region was selectively responsive to human bodies and body parts and termed the region extrastriate body area (EBA). In conclusion, the authors identified a discrete area selective for body perception using fMRI [16]. 

Let us ask a more challenging question: *Can we establish the causal role of these areas in body perception with the help of fMRI?* This is not possible, as fMRI is not a direct measurement of neural activity rather a measurement of blood oxygen level dependent (BOLD) signal which is a proxy for neural activity. This renders the evidence correlational a. Another limitation is the temporal and spatial resolution in relation to our question. The temporal resolution of fMRI is in the order of seconds [17]. Despite these limitations, fMRI offers the finest spatial resolution in an awake human noninvasively. Therefore, fMRI plays a role in exploratory studies that can help identify the regions of interest [18].

This brings us to the question; *What techniques can help establish the causal role of body patches?* From the time of Broca to the present, human lesion studies have stood the test of time as they provide strong causal evidence. As compared to Broca’s era, we have neuroimaging that enables visualization of the brain in vivo. Given the advent of neuroimaging and large imaging datasets, we can conduct group studies rather than single patient studies, which confers statistical validity [19]. Furthermore, computer vision algorithms facilitate faster analysis of group studies involving neuroimaging data [20]. While numerous patients have brain lesions, it is difficult to find subjects with discrete lesions in the EBA. A case report describes a patient with bilateral lesions in the occipitotemporal cortex who developed deficits in movement perception, but unfortunately, no tests were conducted to assess body perception [21]. Apart from this, natural lesions such as stroke offer no experimental control over the size and location of the lesion and they some cortical regions are more frequently involved than the other regions [11].

In the progression from natural to artificial lesions, we would like to draw parallels with the past. Similar to the electrical microstimulation performed by Gustav Fritsch, Eduard Hitzig and Wilder Penfield, modern intracranial electrical microstimulation (iEM) can create precise artificial lesions [22]. By creating these artificial lesions, we can test causality the role of brain regions in perception and behavior [23]. In the clinical setting, iEM can be conducted when the patients receive intracranial EEG (iEEG). iEEG includes both electrocorticography conducted with subdural electrodes and stereo EEG conducted via depth electrodes. Typically, iEEG is conducted to passively record neural activity to confirm seizure foci; however, this setup can be used to conduct iEM studies wherein current can be delivered and artificial lesions can be created [22]. It has an excellent spatial and temporal resolution and as the subjects are typically conscious, there is a direct reporting of perceptual experience rather than having to draw inferences about the experience. This offers unique opportunity to study human cognitive functions [24].

Despite these advantages, there is little mechanistic understanding of how iEM works, that is, whether it causes neuronal excitation, inhibition, or both. Additionally, it is unclear if the current extends further than targeted area and if it influences neighboring regions or passing fibers. Therefore, despite presenting strong causal evidence, one must exercise caution while drawing inferences from iEM studies [25,26]. Another limitation is that the electrode placement is dictated by the clinical presentation, which results in sparse sampling. The areas with high seizure frequency, including the medial temporal lobe, hippocampus, and amygdala, are more readily accessible, whereas other regions are not typically targeted in iEEG and, therefore are not accessible for iEM studies [22]. Lastly, given the highly invasive nature of the technique, human iEM studies for purely research purposes is not viable. As of yet, no study has examined the causal role of body patches using iEM in humans.

In conclusion, lesion studies, both natural and artificial, offer strong causal evidence in structure-function relationship. However, both natural and artificial lesion studies (iEM here) entail studying a diseased brain, thereby endangering the generalizability of inferences [19,22].

This leads us to the next question: *Can we test the causal role of body patches in healthy humans?* This is possible with transcranial magnetic stimulation (TMS), a noninvasive technique that is safe for humans [27]. It is based on Faraday’s principle of electromagnetic induction, which states that an electric current flowing through a coil generates a time-varying magnetic field, which in turn induces an electric current in a conductor in its vicinity [28]. In the case of TMS, a coil is placed near the subject’s scalp which produces a time-varying magnetic field. This induces a small current in the brain and causes a “virtual lesion” [29]. Such an effect can be induced either by a single pulse or by a series of high-frequency stimuli termed as repetitive transcranial magnetic stimulation (rTMS) [30].

**Figure 8:**
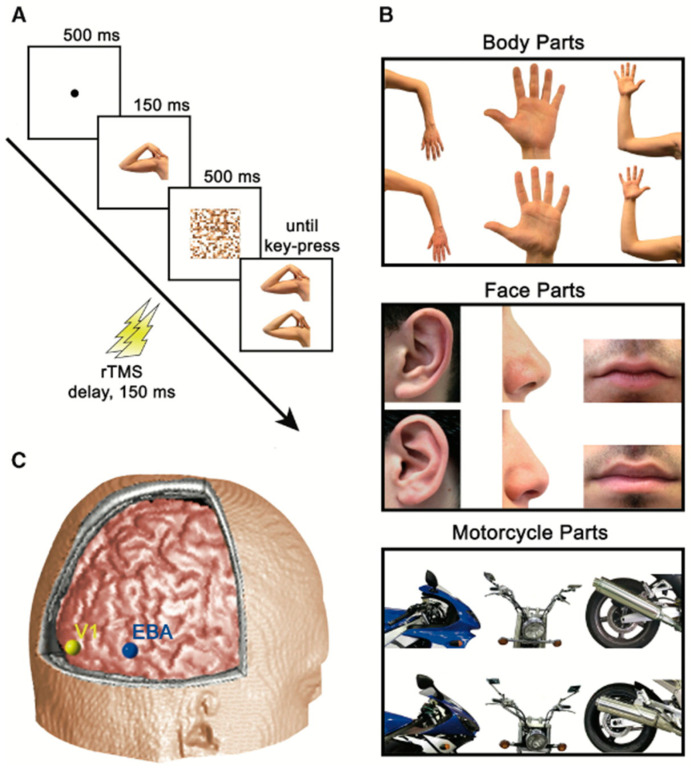
The experiment conducted by Urgesi et al. wherein panel (**A**) depicts rTMS being delivered after 150 ms of stimulus presentation, panel (**B**) shows stimulus categories, and panel (**C**) displays the positions of stimulation sites which include EBA (shown in blue), and primary visual cortexV1 (shown in yellow). Panels (**A**–**C**) adapted with permission from Urgesi et al. (2004).

Since superficial regions of the brain are easily accessible for disruption by rTMS [30], a virtual lesion can be created in EBA. As represented in Figure 8, Urgesi et al. targeted the EBA with rTMS and found that the subjects had difficulties with body-part-related tasks [31].

The fusiform body area (FBA) is another region that is implicated in body perception; however, it is not accessible to rTMS due to its deep location [30]. This is one of the limitations of rTMS studies. Moreover, rTMS is agnostic to neuron subtype and circuitry, thereby limiting neural specificity. Furthermore, it can affect overlaying superficial regions and passing fibers. Therefore, it is challenging to ascertain whether the observed effects are due to disruption in the targeted region or passing fibers [32]. 

In summary, TMS can create reversible virtual lesions in healthy humans. However, the causal inferences that can be drawn from TMS studies are limited due to inadequate spatial precision for deeper brain regions, and poor mechanistic understanding of TMS [30,32].

**Figure 9:**
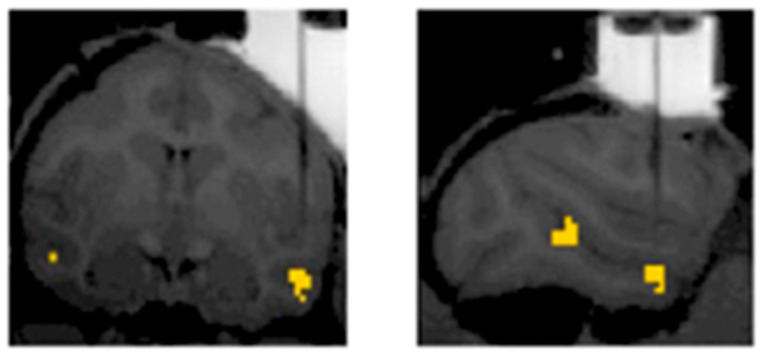
Electrophysiological recordings in non-human primates guided by fMRI. Adapted with permission from Kumar et al. (2019). BOLD activations in yellow indicate differences in BOLD responses evoked by the images of monkey bodies and objects. A vertical shadow indicates an electrode aimed at the ASB.

Next, we ask: *What is the nature of body representations, and how do these representations transform as information progresses in the brain?* To answer this, a combination of neuroimaging and electrophysiological techniques is required. Such a study was reported in 2019 which involved macaques as subjects [33]. The first step was to identify the areas of the brain that responded selectively to macaque body stimuli using fMRI, which were termed body patches. Body patches are fMRI-defined cortical regions in the superior temporal sulcus (STS) which respond selectively to bodies. There are at least two such regions identified in the STS of macaques [34]. These are termed MSB and ASB which stand for mid and anterior STS body patches, respectively. These regions are depicted in yellow in Figure 9. 

An MRI-compatible guide tube was then used to insert electrodes to record local field potentials (LFPs) and multi-unit activity (MUA) from these regions. LFPs and MUAs represent the extracellular activity of an ensemble of neurons [35,36]. The recordings from both MSB and ASB revealed category selectivity for bodies. Another interesting finding in this study was its insight into visual information’s transformation as it traverses the ventral visual stream [33]. Specifically, MSB neurons displayed greater sensitivity to body versus non-body categorization, while ASB neurons exhibited a viewpoint-invariant preference for posture and identity. This could imply that the posteriorly located MSB neurons determine whether the stimulus is an animal or not and that the anteriorly located ASB neurons integrate that information and further process features such as posture and identity [33]. A major advantage of electrophysiological studies is their exceptional spatial and temporal resolution [37]. Since this technique is invasive, it has only been tested on nonhuman primates. When studying cognition, this is critical to consider as unlike humans, nonhuman primates cannot verbally report their perceptions, so the perception is inferred from their behavior. Another limitation of this technique is it is agnostic to neural type and neural circuitry [26]. 

In recent years, optogenetics has evolved as a way to control cellular activity in diverse sets of cells, circuits, and structures of the brain using light. In this technique, opsins which are photoreceptor ion channels are introduced into the neurons of interest via viral vectors [38]. This imparts light sensitivity to the neurons and allows their optical modulation by causing a flux of ions to generate depolarization or hyperpolarization [39]. As light is the effector system, this technique is fully reversible and offers an unsurpassed temporal resolution [40]. Given the precise temporal specificity, this is a suitable technique to study cognition and behavior. Additionally, optogenetics allows cell type-specific tagging in diverse sets of cells, circuits, and brain structures. Inhibitory or excitatory neuron populations, for example, can be selectively manipulated [41]. Optogenetics has widely been used in mouse models and has now been extended to non-human primates to examine long-standing technical and conceptual questions in systems neuroscience [42]. There is, however, a risk of severe toxicity when adeno-associated virus (AAV) is used in high vector copy numbers for optogenetics for large brain areas in higher primates including humans [43]. The recent development of engineered AAVs, however, shows promise by providing non-invasive gene delivery vectors with minimal systemic toxicity to rodents and non-human primate nervous systems [44].

## 4. The Future: Clinical and Basic Neuroscience Implications

In this review, we have discussed the exciting journey of cognitive neuroscience from phrenology to optogenetics. Neural correlates of behavior can be studied at various levels—the single neurons, ensembles of neurons, circuits, and systems. Among these, understanding circuit dynamics has been identified as having the potential to transform the field [45]. Multiple circuit-level monogenic animal disease models of the various behavioral and neuropsychiatric conditions exist [46]. These monogenic animal models offer not only an insight into how the diseased brain has atypical structural and functional dynamics, but also a window for studying the effect of interventions and plasticity [46]. Furthermore, it is imperative that we make efforts to improve these techniques in order to make them practical and safe for use in primates in the future.

Lesion localization will become more precise in humans with the advent of (1) large neuroimaging datasets in clinical and basic research and (2) improved analysis techniques such as voxel-based lesion-symptom mapping (VLSM) and deep learning. VLSM studies entail the categorization of the subjects either based on lesions or behavioral deficits. Thereafter, the group is compared on a voxel-by-voxel basis [47,48]. VLSM studies have produced accurate localization data for stroke patients with deficits in cognition [49], language [50], memory [51] and executive functions [52]. VLSM and deep learning techniques can be used in conjunction to get a better understanding of brain-behavior relationships by combining them with large datasets [20].

Applying Marr’s level of an information processing unit to neural circuitry-behavioral relationship, the complete description of the circuits involved in cognition and behavior requires three levels of understanding, which include an understanding of the why, what, and how aspects of an information processing system functions [4]. At the computational level, we will require to first characterize why the function in question is being carried out and what relevance it holds for the subject. At the algorithmic level, we will need to better characterize the qualities of individual neurons and their synaptic and supplementary communications with other neurons. Moreover, at this level, we will need to elucidate how the information transforms through the circuit and results in behavior. Finally, at the implementation level, we require to be able to implement the proposed circuit either naturally or artificially. 

A multidisciplinary approach is needed for characterizing fundamental brain functions [53]. This includes integration across fields such as data science, neuroimaging, and engineering; and bringing together inferences of both human and non-human models [45]. This integrative approach will require widespread dataset and data analysis sharing [45]. The advancements in the characterization of cognitive functions will ultimately progress our understanding of the fundamental brain processes [45]. This in turn will lay the foundation for better diagnosis, management, and finally treatment and reversal of the diseased brain states.

## Data Availability

Not applicable.
